# Comparison of an inflammation-based prognostic score (GPS) with performance status (ECOG) in patients receiving platinum-based chemotherapy for inoperable non-small-cell lung cancer

**DOI:** 10.1038/sj.bjc.6601789

**Published:** 2004-04-13

**Authors:** L M Forrest, D C McMillan, C S McArdle, W J Angerson, D J Dunlop

**Affiliations:** 1 University Department of Surgery, Royal Infirmary, Glasgow G31 2ER, UK; 2 Department of Medical Oncology, Royal Infirmary, Glasgow G31 2ER, UK

**Keywords:** systemic inflammatory response, prognostic score, stage, performance status, survival, chemotherapy, non-small-cell lung cancer

## Abstract

The value of an inflammation-based prognostic score (GPS) was compared with performance status (ECOG) in patients (*n*=109) receiving platinum-based chemotherapy for inoperable non-small-cell lung cancer. On multivariate analysis with ECOG, white cell count and the GPS entered as covariates, only the GPS was a significant independent predictor of survival (HR 1.88, 95% CI 1.25–2.84, *P*=0.002).

Non-small-cell lung cancer (NSCLC) is the most common cause of cancer death in North America and Western Europe. Most patients present with advanced inoperable disease; the prognosis of these patients is extremely poor. In selected patients, platinum-based regimens have been shown to have a beneficial but modest impact on survival (Klastersky and Paesmans, 2001). Conventionally, the selection of patients for chemotherapy has been based on clinico-pathological criteria, including age, stage and performance status (Numico *et al*, 2001).

There is increasing evidence that the presence of a systemic inflammatory response, as evidenced by elevated circulating concentrations of C-reactive protein concentrations, is associated with early recurrence and poor survival, independent of stage, in a variety of common solid tumours (Ikeda *et al*, 2003; McMillan *et al*, 2003). In advanced disease, an elevated C-reactive protein has also been shown to associated with poor survival (O’Gorman *et al*, 2000; Mahmoud and Rivera, 2002).

Furthermore, in an unselected cohort of patients with inoperable NSCLC, the Glasgow Prognostic Score (GPS), a cumulative prognostic score based on C-reactive protein and albumin, had similar prognostic value to that of stage and performance status (Forrest *et al*, 2003). The question of whether the GPS would be useful in the selection of appropriate treatment for patients with inoperable NSCLC remains to be determined.

The aim of the present study was to assess the value of the GPS in patients receiving chemotherapy for inoperable NSCLC.

## MATERIALS AND METHODS

### Study design

Patients presenting with inoperable NSCLC (stages III and IV) to a single multidisciplinary oncology clinic in Glasgow Royal Infirmary between March 2000 and June 2003 were studied prospectively. All patients had cytologically or histologically confirmed disease and were staged on the basis of clinical findings, chest X-ray and, where appropriate, bronchoscopy, liver ultrasound, isotope bone scan and computerised tomography of the thorax, according to the American Thoracic Society TNM classification (Mountain, 1991).

Clinical stage, tumour type and performance status (Eastern Cooperative Oncology Group, ECOG) were recorded at the time of diagnosis. A blood sample was also obtained for the measurement of white cell count, albumin and C-reactive protein concentrations. Patients received between one and six cycles of platinum-based chemotherapy.

The study was approved by the Research Ethics Committee of Glasgow Royal Infirmary.

### Methods

Blood parameters: Routine laboratory measurements of albumin and C-reactive protein concentration were carried out. The coefficient of variation for these methods, over the range of measurement, was less than 5% as established by routine quality control procedures.

The GPS was constructed as previously described (Forrest *et al*, 2003). Briefly, patients with both an elevated C-reactive protein (>10 mg l^−1^) and hypoalbuminaemia (<35 g l^−1^) were allocated a score of 2. Patients in whom only one of these biochemical abnormalities was present were allocated a score of 1. Patients in whom neither of these abnormalities was present were allocated a score of 0.

### Statistics

Univariate survival analysis was performed using the Kaplan–Meier method with the log-rank test. Multivariate survival analysis and calculation of hazard ratios (HR) were performed using Cox regression analysis. Deaths up to 31st October 2003 were included in the analysis. Analysis was performed using SPSS software (SPSS Inc., Chicago, IL, USA).

## RESULTS

The characteristics of patients with inoperable NSCLC receiving platinum-based chemotherapy (*n*=109) are shown in Table 1

**Table 1:** Clinical characteristics and survival in patients with inoperable NSCLC receiving platinum-based chemotherapy: univariate survival analysis

	**Patients**	**Survival (months)**	***P*-value**
	109 (100%)	Median (95% CI)	
*Age*			
<60 years	41 (38)	10.6 (3.8–17.3)	
⩾60 years	68 (62)	13.5 (11.2–15.9)	0.681
			
*Sex*			
Male	63 (58)	15.1 (11.0–19.2)	
Female	46 (42)	10.6 (10.1–11.1)	0.433
			
*Stage*			
III	52 (47)	13.5 (9.1–18.0)	
IV	57 (52)	12.2 (7.8–16.6)	0.337
			
*Type*			
Squamous	40 (37)	15.1 (7.9–22.4)	
Adenocarcinoma	46 (42)	14.0 (9.5–18.6)	
Other	23 (21)	11.7 (9.7–13.6)	0.192
			
*Performance status (ECOG)*			
0	29 (27)	16.0 (9.2–22.7)	
1	71 (65)	12.2 (9.9–14.5)	
2	9 (8)	7.1 (1.7–12.4)	0.405
			
*White cell count*			
<10 × 10^9^/l	62 (56)	16.6 (13.1–20.1)	
≥10 × 10^9^/l	47 (44)	11.7 (9.4–14.0)	0.029
			
*GPS*			
0	27 (25)	17.0 (14.0–19.9)	
1	69 (63)	12.1 (10.0–14.1)	
2	13 (12)	7.1 (4.9–9.2)	0.005

. The majority were male and over the age of 60 years. Approximately 50% had stage III disease, 90% had an ECOG performance status of 0–1, 75% had an elevated C-reactive protein and 10% had hypoalbuminaemia. The majority (68%) received cisplatin-based chemotherapy and the remainder carboplatin-based chemotherapy.

**Figure 1:**
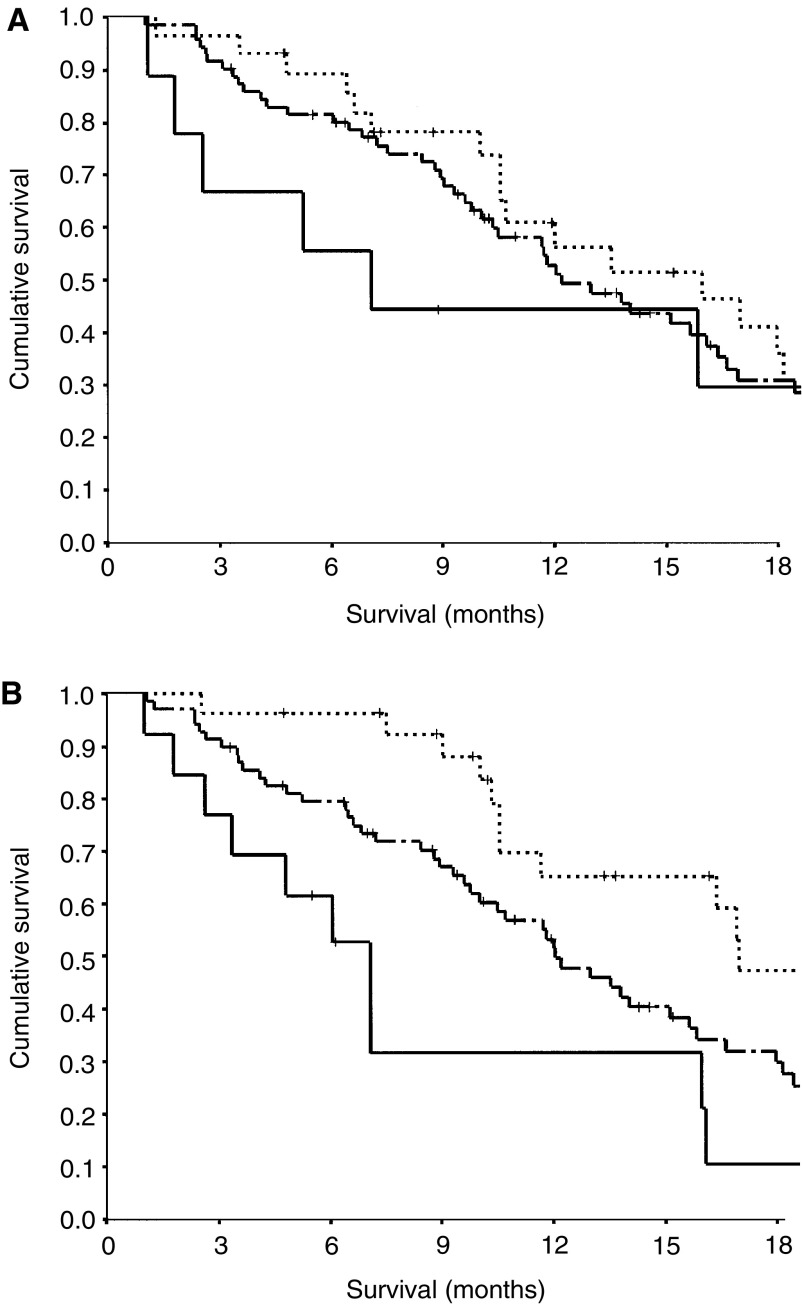
(**A**) The relationship between ECOG performance status (0 ……., 1 —–, 2 _____) in patients with inoperable non-small-cell lung cancer receiving platinum-based chemotherapy. (**B**) The relationship between the GPS (0 ……., 1 —–, 2 _____) in patients with inoperable non-small-cell lung cancer receiving platinum-based chemotherapy.

In total, 71 (65%) of patients died during the follow-up period. On univariate survival analysis, both white cell count and GPS were significant predictors of survival. Median survival times in the groups with an ECOG of 0, 1 and 2 were 16, 12 and 7 months, respectively, but were associated with wide confidence intervals and the difference in survival was not significant (Figure 1A

). Median survival times in the groups with a GPS of 0, 1 and 2 were 17, 12 and 7 months respectively (*P*<0.01, Figure 1B).

On multivariate analysis with ECOG, white cell count and the GPS entered as covariates, only the GPS was a significant independent predictor of survival (HR 1.88, 95% CI 1.25–2.84, *P*=0.002).

## DISCUSSION

Conventionally, in patients with inoperable NSCLC, the decision whether or not to offer chemotherapy is primarily based on performance status. However, the assessment of performance status is subjective. For example, significant differences in the assessment of performance status have been reported between oncologists, nurses and patients, oncologists being the most optimistic in their assessment and patients the least (Ando *et al*, 2001). As a result there is continuing interest in the development of prognostic scores, which better reflect clinical outcome (Bennett and Ryall, 2000; Sloan *et al*, 2001).

In the present study, an inflammation-based prognostic score based on standard laboratory measurements, the GPS, appeared to be superior to performance status in predicting outcome following platinum-based chemotherapy. This may be in part because the assessment of performance status reflects functional status at a specific point in time. In contrast, the GPS, based as it is on the presence of an ongoing systemic inflammatory response and hypoalbuminaemia, predicts the progressive nutritional decline of the patient (McMillan *et al*, 2001; Scott *et al*, 2002). Indeed, it has long been recognised that progressive weight loss is associated with poor tolerance to chemotherapy (Chlebowski *et al*, 1996; Paesmans *et al*, 1997).

More recently, it has been reported that cytochrome *P*450 3A activity, the principal drug metabolising enzyme in a variety of chemotherapeutic agents, is compromised in advanced lung cancer patients with an elevated C-reactive protein concentration (Rivory *et al*, 2002; Slaviero *et al*, 2003). One might therefore postulate that the presence of a systemic inflammatory response would be associated with increased toxicity in patients receiving platinum-based chemotherapy. It was therefore of interest that 40% of patients with a GPS of 0 received six cycles platinum-based chemotherapy compared with only 9% of those with a GPS of 1 or 2 (*P*=0.003, Fisher's exact test). This suggests that the presence of a systemic inflammatory response may be an important factor in determining the ability of patients to tolerate platinum-based chemotherapy.

It is possible that in patients with inoperable non-small-lung cancer, an elevated C-reactive protein concentration might reflect intercurrent infection. If this were the case it might be expected that the increase in circulating C-reactive protein concentrations would be associated with a rise in the white cell count. However, in the present study, although the white cell count was significantly correlated with C-reactive protein concentrations, the magnitude of the relationship was small (*r*^2^=10.6%). This would suggest that infection was not the main stimulus to the increased C-reactive protein concentrations.

The results of the present study suggest that the GPS offers additional prognostic information that may assist in the selection of appropriate patients with inoperable NSCLC for platinum-based chemotherapy.
